# Efficient experimental design and analysis strategies for the detection of differential expression using RNA-Sequencing

**DOI:** 10.1186/1471-2164-13-484

**Published:** 2012-09-17

**Authors:** José A Robles, Sumaira E Qureshi, Stuart J Stephen, Susan R Wilson, Conrad J Burden, Jennifer M Taylor

**Affiliations:** 1 CSIRO Plant Industry, Black Mountain Laboratories, Canberra, Australia; 2 Mathematical Sciences Institute, Australian National University, Canberra, Australia; 3 Prince of Wales Clinical School and School of Mathematics and Statistics, University of New South Wales, Sydney, Australia

**Keywords:** RNA-Seq, Differential expression analysis, Sequencing depth, Replication, Experimental design, Multiplex

## Abstract

**Background:**

RNA sequencing (RNA-Seq) has emerged as a powerful approach for the detection of differential gene expression with both high-throughput and high resolution capabilities possible depending upon the experimental design chosen. Multiplex experimental designs are now readily available, these can be utilised to increase the numbers of samples or replicates profiled at the cost of decreased sequencing depth generated per sample. These strategies impact on the power of the approach to accurately identify differential expression. This study presents a detailed analysis of the power to detect differential expression in a range of scenarios including simulated null and differential expression distributions with varying numbers of biological or technical replicates, sequencing depths and analysis methods.

**Results:**

Differential and non-differential expression datasets were simulated using a combination of negative binomial and exponential distributions derived from real RNA-Seq data. These datasets were used to evaluate the performance of three commonly used differential expression analysis algorithms and to quantify the changes in power with respect to true and false positive rates when simulating variations in sequencing depth, biological replication and multiplex experimental design choices.

**Conclusions:**

This work quantitatively explores comparisons between contemporary analysis tools and experimental design choices for the detection of differential expression using RNA-Seq. We found that the DESeq algorithm performs more conservatively than edgeR and NBPSeq. With regard to testing of various experimental designs, this work strongly suggests that greater power is gained through the use of biological replicates relative to library (technical) replicates and sequencing depth. Strikingly, sequencing depth could be reduced as low as 15% without substantial impacts on false positive or true positive rates.

## Background

RNA sequencing (RNA-Seq) allows an entire transcriptome to be surveyed at single-base resolution whilst concurrently profiling gene expression levels on a genome scale [1]. RNA-Seq is an attractive approach as it profiles the transcriptome directly through sequencing and therefore does not require prior knowledge of the transcriptome under consideration. An example of the use of RNA-Seq as a high-resolution exploratory tool is the discovery of thousands of additional novel coding and noncoding genes, transcripts and isoforms of known genes despite the prior extensive annotation of the mouse [2-4] and human genomes [5,6].

Arguably, the most popular use of RNA-Seq is profiling of gene expression or transcript abundance between samples or differential expression (DE). The efficiency, resolution and cost advantages of using RNA-Seq as a tool for profiling DE has prompted many biologists to abandon microarrays in favour of RNA-Seq [7,8].

Despite the advantages of using RNA-Seq for DE analysis, there are several sources of sequencing bias and systematic noise that need to be considered when using this approach. Clearly, RNA-Seq analysis is vulnerable to the general biases and errors inherent in the next-generation sequencing (NGS) technology upon which it is based. These errors and biases include: sequencing errors (wrong base calls), biases in sequence quality, nucleotide composition and error rates relative to the base position in the read [9,10], variability in sequence depth across the transcriptome due to preferential sites of fragmentation, variable primer and transcript nucleotide composition effects [11] and finally, differences in the coverage and composition of raw sequence data generated from technical and biological replicate samples [1,12].

Recently, there have been several investigations [13-15] into the biases that affect the accuracy with which RNA-Seq represents the absolute abundance of a given transcript as measured by high precision approaches such as Taqman RT-PCR [16]. It has been shown that these abundance measures are prone to biases correlated with the nucleotide composition [14,17] and length of the transcript [1,18]. Several within and between sample correction and normalisation procedures have recently been developed to address these biases either as nucleotide composition effects [17] or various combinations of nucleotide, length or library preparation biases [14,15]. These approaches all yield improvements in the correspondence of RNA-Seq read counts with expression estimates gained by other experimental approaches.

Despite the known biases, RNA-Seq continues to be widely and successfully used to profile relative transcript abundances across samples to identify differentially expressed transcripts [19]. The profile of a given transcript across a biological population would be hoped to be less prone to nucleotide composition and length biases as these variables remain constant. Nevertheless, to accurately detect DE across samples it is necessary to understand the sources of variation across technical and biological replication and where possible respond to these with an appropriate experimental design and statistically robust analysis [17,20]. To date, there has been little discussion in the literature of efficient experimental designs for the detection of DE and a lack of consensus about a standard and comprehensive approach to counter the many sources of noise and biases present in RNA-Seq has meant that some of the biological community remain sceptical about its reliability and unsure of how to design cost-efficient RNA-Seq experiments (see [19]).

Good experimental design and appropriate analysis is integral to maximising the power of any NGS study. With regard to RNA-Seq, important experimental design decisions include the choice of sequencing depth and number of technical and/or biological replicates to use. For researchers with a fixed budget, often a critical design question is whether to increase the sequencing depth at the cost of reduced sample numbers or to increase the sample size with limited sequencing depth for each sample [20].

### Sequencing depth

Sequencing depth is usually referenced to be the expected mean coverage at all loci over the target sequence(s), in the case of RNA-seq experiments assuming all transcripts having similar levels of expression. Without the benefit of extensive previous RNA-Seq studies, it is difficult in most cases to estimate prior to data generation the optimal sequencing depth or amount of sequencing data required to adequately power the detection of DE in the transcriptome of interest. Pragmatically, RNA-seq sequencing depth is typically chosen based on an estimation of total transcriptome length (bases) and the expected dynamic range of transcript abundances. Given the dynamic nature of the transcriptome, the suitability of these estimates could vary substantially across organisms, tissues, time points and biological contexts.

Wang et al. [21] found a significant increase in correlation between gene transcripts observed and number of sequence reads generated when increasing sequencing depth from 1.6 to 10 million reads after which the gains plateau – 10 million reads detected about 80% of the annotated chicken transcripts. Despite the expectation of continuous sequencing depth increases in the near future, Łabaj et al. [22] argue that most of the additional reads will align to the subset of already extensively sampled transcripts. As a result, transcripts with low to moderate expression levels will remain difficult to quantify with good precision using current RNA-Seq protocols even at higher read depths. Greater sequencing depth will also increase sensitivity to detect smaller changes in relative expression, however this does not guarantee that these changes have functional impact in the biological system under study as opposed to tolerated fluctuations in transcript abundance [20]. Ideally, an efficient experimental design will be informed by an understanding of when increasing sequencing depth begins to provide rapidly diminishing returns with regard to transcript detection and DE testing.

### Replication

Replication is vital for robust statistical inference of DE. In the context of RNA sequencing, multiple nested levels of technical replication exist depending upon whether it is the sequence data generation, library preparation or RNA extraction technical processes that are being replicated from the same biological sample. Several published studies have incorporated technical replicates into their RNA-Seq experimental designs [23-25]. The degree of technical variation present in these datasets appears to vary and the main source of technical variation appears to be library preparation [15]. Biological replication measures variation within the target population and simultaneously can counteract random technical variation as part of independent sample preparation [20].

It has been shown that power to detect DE improves when the number of biological replicates *n* is increased from *n *= 2 to *n *= 5 [26], however, to date few studies have incorporated extensive biological replication and extensive testing of the effects of replication on power is needed. More recently with the increasing utility and availability of multiplex experimental designs, the incorporation of biological replicates with decreased sequencing depth is becoming a much more attractive and cost-effective strategy. The relative merits of sacrificing sequencing depth for increased replication has not been rigorously explored.

### Efficient experimental design

Multiplexing is an increasingly popular approach that allows the sequencing of multiple samples in a single sequencing lane or reaction and consequently the reduction in sequencing costs per sample [27,28]. Multiplexing uses indexing tags, “barcodes” or short (≤ 20 bp) stretches of sequence that are ligated to the start of sample sequence fragments during the library preparation step. Barcodes are distinct between sample libraries and allow pooling for sequencing followed by allocation of reads back to individual samples after sequencing by analysis of the sequenced barcode. Multiplex barcode designs are routinely available with up to 12 samples in the same lane, recently up to 96 yeast DNA samples were profiled in single lane [28]. Novel methods are continuing to emerge for low-cost strategies to multiplex RNA-Seq samples [29]. With the dramatic increases in sequencing yields being achieved with current chemistries and new platforms, multiplexing is becoming the method of choice to increase sample throughput. These designs have direct impacts on sequencing depth generated that need to be considered in the power of the experimental design. Also, when multiplex strategies are used, biologists need to be mindful of potential systematic variations between sequencing lanes. These variations can be addressed through randomisation or blocking designs to distribute samples across lanes, see [30] for a discussion of barcoding bias in multiplex sequencing, and [31] for an alternative to barcoding. In a comparison between microarray and NGS technologies in synthetic pools of small RNA, Willenbrock et al. [13] found that multiplexing resulted in decreased sensitivity due to a reduction of sequencing depth and a loss of reproducibility; however the authors did not investigate power for detection of DE in their study.

### Approach

Improving detection of DE requires not only an appropriate experimental design but also a suitably powered analysis approach. Several algorithms have recently been developed specifically to appropriately handle expected technical and biological variation arising from RNA-Seq experiments. A non-exhaustive list of these algorithms is: edgeR [32], DESeq [25], NBPSeq [33], BBSeq [34], FDM [35], RSEM [36], NOISeq [37], Myrna [38], Cuffdiff [2]. A thorough comparison of these packages’ performance with datasets of different properties falls beyond the scope of this study, however before considering issues relating to power and experimental design, it is important to investigate whether packages for DE analysis give the correct type I error rate under the null hypothesis of no DE. To do this evaluation we considered three popular packages for DE analysis of RNA-sequencing data. These packages are based on a negative binomial distribution model of read counts [39] and include edgeR [32], DESeq [25] and NBPSeq [33].

To quantify the effects of different sequencing depths and replication choices we compared a range of realistic experimental designs for their ability to robustly detect DE. Using simulated data with known DE transcripts allowed us to estimate the false positive rate (FPR) and true positive rate (TPR) of DE calls. The changes of these rates were used to compare the detection power yielded by each choice of number of biological replicates and sequencing depth.

In the Methods section, we outline the definitions used for FPR and TPR as well as explaining the method used for the construction of the synthetic data; which includes induced differential expression, simulates the variations that biological replicates introduce and simulates loss of sequencing depth.

In our study, we test a wide range of real-world experimental design scenarios for performance under the null hypothesis and in the presence of DE. In these scenarios both the numbers of biological replicates *n* and the sequencing depth are varied. This provides a comprehensive quantitative comparison of different experimental design strategies and is particularly informative for those accessing modern multiplex approaches.

## Results

### Comparisons of statistical methods: edgeR, DESeq, and NBPSeq using simulated data under the null

**Figure 1:**
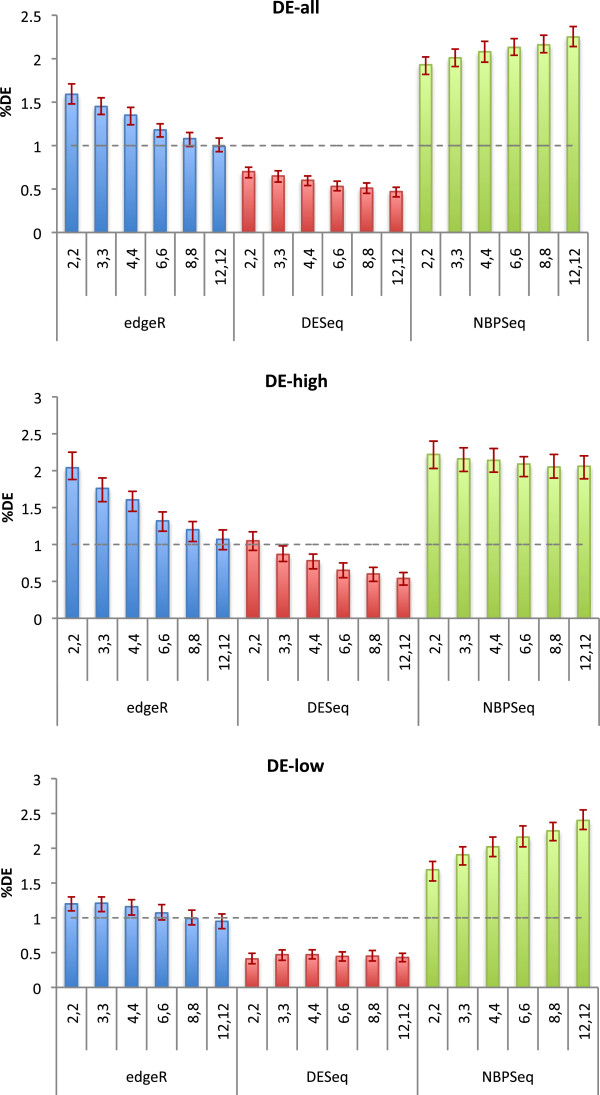
**The percentage of transcripts reported differentially expressed, FPR defined by Eq. 4 by three software packages for synthetic data generated under the null hypothesis of no DE between two conditions.** In the lower two panels the set of transcripts has been divided into those with greater than 100 counts (DE-high) and those with less than or equal to 100 counts (DE-low) averaged over biological replicates. The number of biological replicates in each condition was varied over the range *n *= 2, 3, …12. The experiment was repeated for 100 independently generated datasets. The top of each bar is the median value obtained and its 90% confidence interval.

**Figure 2:**
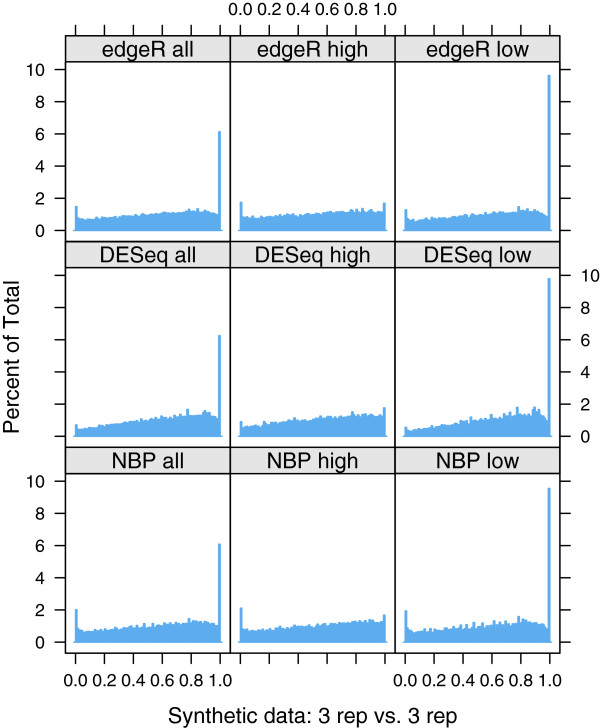
**Histograms of p-values calculated by three software packages for one particular example of synthetic data generated under the null hypothesis for the case *n *= 3.** In the two right hand columns the set of transcripts has been divided into high-count transcripts (> 100 counts) and low-count transcripts (≤ 100 counts) respectively. ‘Percentage of total’ is the percentage of p-values falling within each of 100 bins in each histogram.

To test the performance of each package under the null hypothesis, we simulated sets of *n* “control” and *n* “treatment” lanes of counts in accordance with the procedure described in the Methods section, for a range of values of *n* and with no DE between treatments. For each value of *n* and for each package the simulation and testing were repeated 100 times. Figure 1 shows the percentage of transcripts reported as differentially expressed at the 1% significance level by each of the three packages for a range of values of *n*. The height of each bar is the median value obtained from 100 repetitions of the synthetic data generation with its associated 90% confidence intervals. Under the null hypothesis, the percentage reported is the false positive rate (FPR) defined by Eq. 4, and should match the significance level of *α *= 1*% *if the package is performing correctly. Also shown are FPRs for high-count transcripts (> 100 counts averaged across biological replicates) and low-count transcripts (≤ 100 counts averaged across biological replicates). Figure 2 shows an example of the p-value distribution obtained for one experiment at *n *= 3 biological replicates. Ideally, p-values should have a uniform distribution in the interval [0,1] if the package is performing correctly.

Immediately noticeable in the p-value histogram is a sharp spike in the right hand bin for low count transcripts, which is observed to be present in general for all values of *n* and all packages. This is a known artifact of calculating p-values for discrete random variables using the method described in [40] and summarised in our Methods subsection ‘Under the null hypothesis’: when count sums in both conditions are equal the computed p-value is exactly 1. The situation is most likely to occur for transcripts with extremely low counts, in which case it is difficult to draw meaningful conclusions regarding DE via any method. The behaviour at the left hand end of the histogram, which drives the FPRs plotted in Figure 1, varies considerably between packages and numbers of biological replicates. It is affected mainly by the method used for estimating a dispersion parameter *ϕ*_*i*_ for each transcript *i* (see Methods section).

The package edgeR performs well for large numbers of biological replicates (*n *= 12), for which squeezing of the dispersion estimate towards the common dispersion is minimal, and a tagwise estimate is appropriate. For small numbers of biological replicates, because the dispersion cannot be estimated accurately on a per-transcript basis, information is borrowed from the complete set of transcripts to squeeze the estimate towards a common dispersion estimate. For the high-count transcripts in particular, the squeezing causes the dispersion of the most highly dispersed transcripts to be underestimated, causing too many transcripts to be deemed differentially expressed, leading to an inflated FPR.

In an effort to be conservative, DESeq chooses as its estimate of dispersion the maximum of a per-transcript estimate and the functional form Eq. 2 which is fitted to the per-transcript estimates for all transcripts. Our results indicate that the method performs well for the high-count transcripts when the number of biological replicates is small (*n *= 2 or 3), but is otherwise over-conservative. This is generally to be preferred to an inflated FPR, as one has more evidence that what is called DE is truly DE.

The package NBPSeq imposes the functional relationship Eq. 3, which appears to be too restrictive for a number of relatively highly dispersed transcripts. For those transcripts the dispersion parameter is underestimated, leading to an overestimate of significance and hence an inflated FPR irrespective of the number of biological replicates.

Based on these results we selected DESeq (v1.6.1) and edgeR (v2.4.0) for use in subsequent experimental design testing. Throughout these tests, results obtained using DESeq and edgeR are mostly compatible with each other. However, our comparison revealed a slightly inflated FPR from edgeR while DESeq behaves more conservatively throughout. Therefore, in the following section we will focus on the results obtained using DESeq while the analogous results obtained with edgeR are presented in the Additional file 1: Figure S2.

### Comparison of statistical methods: DESeq and edgeR using simulated data with 15% DE transcripts

To test the performance of packages in the presence of an alternate hypothesis, we simulated sets of *n* “control” and *n* “treatment” lanes of counts with 15% of the transcripts either up- or down-regulated according to the procedure described in the Methods section. All results presented from this point on are derived from DESeq.

### Detection of DE as a function of number of biological replicates *n*

With an increase in replication we saw a steady increase in the percentage DE calls by DESeq (call rate), increasing from 0.44% to 5.12% as *n* increased from 2 to 12 (at 100% depth). As *n* increased, the FPR, defined by Eq. 5 at a significance level of *α *= 1*%*, remained below 0.1*%* for all values of *n*, and the TPR, defined by Eq. 6 with *α *= 1*%*, increased substantially from 3.26% to 41.57% (see Table 1).

**Table 1:** Effects of biological replication on power to detect DE using DESeq

**%**	***n = *2**	***n = *3**	***n = *4**	***n = *6**	***n = *8**	***n = *12**
call rate %	0.44	1.15	1.76	3.03	4.08	5.12
FPR %	0.04	0.06	0.06	0.06	0.05	0.04
TPR %	3.26	8.95	13.95	24.30	32.72	41.57

**Figure 3:**
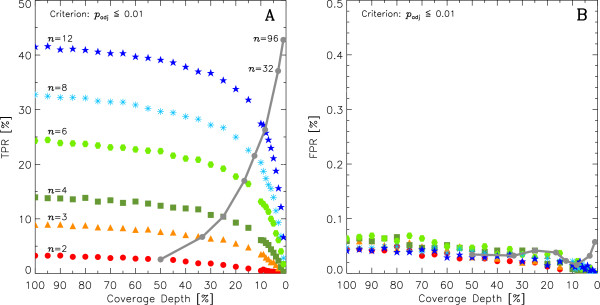
**TPR and FPR detected by DESeq as a function of sequencing depth and replication.** Different symbols represent the number *n* of control vs. treatment samples (*n *= 2, 3, 4, 6, 8, and 12) across sequence depths [100*%*→1*%*]. **A:** TPR (Eq. 6 at *α *= 1*%*) *p*_adj_ ≤ 0.01. **B:** FPR (Eq. 5 at *α *= 1*%*) *p*_adj_ ≤ 0.01. The solid grey line (“multiplex line”) connecting the TPR values of *n* biological replicates at $\frac{1}{n}\times 100\%$ sequencing depth shows the increase of TPR as more biological replicates *n* are used despite the loss power due to the sequencing depth reduction required by the multiplexing of lanes. This trend remains true even for the *n *= 32 and *n *= 96 cases.

Effects of biological replication on power to detect DE using DESeq. FPR and TPR are defined in Eqs. 5 & 6 respectively at 1%. “call rate” is the total number of reported positives / the total number of transcripts. These values are also represented in Figure 3 at 100% sequencing depth.

### Detection of DE as a function of sequencing depth

Figure 3 represents the combined results of decreasing sequencing depth for all values of *n*. It can be seen that as sequencing depth decreases the TPR generated by DESeq decreases monotonically across all *n* while the FPR remains below 0.1*%*(the corresponding results obtained using edgeR are shown in Additional file 1: Figure S2).

Table 2 shows the FPR for all biological replicates *n* and a subset of sequencing depths: 25%, 50%, 75% and 100%, the FPR remains below 0.1*% *at all sequencing depths. Table 3 shows the TPR reported by DESeq for the same subset of sequencing depths, here the TPR increases strongly as sequencing depth increases for any number of biological replicates *n*.

**Table 2:** **Effects of sequencing depth on FPR at different *n *and depths**

**Depth**	***n = *2**	***n = *3**	***n = *4**	***n = *6**	***n = *8**	***n = *12**
25%	0.02	0.02	0.04	0.03	0.03	0.03
50%	0.03	0.03	0.04	0.05	0.04	0.03
75%	0.04	0.06	0.05	0.07	0.04	0.04
100%	0.04	0.06	0.06	0.06	0.05	0.04

Effects of sequencing depth on FPR values for a subset of our tested depths = 25%, 50%, 75% & 100%.

**Table 3:** **Effects of sequencing depth on TPR at different *n *and depths**

**Depth**	***n = *2**	***n = *3**	***n = *4**	***n = *6**	***n = *8**	***n = *12**
25%	1.57	6.24	10.40	19.18	26.08	35.41
50%	2.58	7.63	12.40	22.34	29.66	39.16
75%	3.01	8.47	13.16	23.44	31.57	40.65
100%	3.26	8.95	13.95	24.30	32.72	41.57

Effects of sequencing depth on TPR values for a subset of our tested depths = 25%, 50%, 75% & 100%.

### Detection of DE across multiplex experimental design strategies

**Figure 4:**
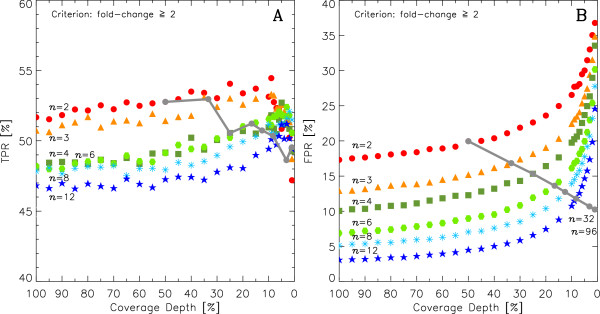
**Same as Figure**3**but using 2-fold-changes as the criterion for FPR and TPR instead of *p***_**adj **_**≤ 0.01. A:** TPR fold-change ≥ 2. **B:** FPR fold-change ≥ 2. The “multiplex line” connects the TPR and and FPR values of *n* biological replicates at $\frac{1}{n}\times 100\%$ sequencing depth.

We simulated various scenarios of multiplexing *n*-control samples vs. *n*-treatment samples into two sequencing lanes – each control and treatment sample at a sequencing depth $=\frac{1}{n}\times 100\%$. In Figures 3 and 4, a solid grey line connecting every value of *n* at its corresponding sequencing depth provides a summary of the performance of these multiplexing scenarios. We call this trend the “multiplex line” and it provides an insight into the results obtained by increasing the number of biological replicates used into a fixed number of sequencing lanes, in this case 2 sequencing lanes.

The multiplex line in Figure 3 shows a clear increase in TPR as replication increases despite the loss of detection power that decreasing sequencing depth induces. It can also be seen that the FPR remains below 0.1*%* for all multiplex scenarios tested (Figure 3B). Note that for completeness we also added multiplex scenarios for *n *= 32 &*n *= 96, whose results follow the trends well. The multiplex line strongly favours adding more biological replicates despite the inherent loss of sequencing depth as shown by its dramatic positive slope for the TPR while maintaining a roughly constant, low FPR.

### Fold-changes as indicators of DE

It is common practice among biologists to use fold-change, rather than p-values, as an indicator of DE. Figure 4 shows results analogous to those of Figure 3 when the criterion of fold-change ≥ 2 (instead of p-values) is used to detect DE: as replication *n* increases, both TPR and FPR decrease because more biological replicates have the effect of averaging out differences between control and treatment lanes. Note that, as sequencing depth decreases, the FPR increases owing to the growing number of transcripts with very low numbers of counts (Figure 4B), in which case the Poisson shot noise of the sequencer can easily induce a spurious doubling or halving of counts. This effect is ameliorated by adding 1 count to all transcripts prior to DE analysis – doing so, does not affect the calculation of p-values (data not shown).

## Discussion

### Comparisons of DE algorithms: edgeR, DESeq and NBPSeq

Our comparison of these three DE detection algorithms under the null hypothesis revealed different performances (measured by their FPR) when different numbers of biological replicates *n*, are used. DESeq consistently performed more conservatively across the different *n* biological replicates scenarios. DESeq’s performance was closest to the expected significance level when only using high-count (counts > 100) transcripts while for only low-count (counts ≤ 100) transcripts over-conservative behaviour is shown. edgeR overestimates DE detection for small values of *n* while its performance improves as *n* increases. edgeR’s level of detection is constant over *n* when only low-count transcripts are used while overestimation increases when only high-count transcripts are used. NBPSeq overestimated detection across *n* for the three scenarios (all-transcripts, high-counts and low-counts).

This comparison led us to use both DESeq and edgeR throughout our replication and sequencing depth simulations. We ultimately chose DESeq’s results^a^ as this package behaved slightly more conservatively and appeared less sensitive to changes in replication (see Figure 1). In a study by Tarazona et al. [37], it is argued that negative-binomial based DE analysis packages like DESeq and edgeR are highly sensitive to sequence depth increases and are therefore unable to control the FPR as sequencing depth increases. Tarazona et al. propose a non-parametric algorithm (NOISeq) to calculate DE based on a noise distribution created with fold-changes and the absolute differences between the transcript’s control and experiment lane counts. However, Kvam and Liu [26] argue that due to the small number of replicates typically used for RNA-Seq experiments, non-parametric methods do not offer enough detection power and suggest that current statistical methods to detect DE genes based on parametric models for RNA-Seq data (e.g. DESeq and edgeR) remain a more adequate approach. In our study we also find that both DESeq and edgeR tend to slightly increase the FPR as sequencing depth increases – as higher depths induce DESeq and edgeR to assign smaller p-values to transcripts with small fold-changes^b^. However in no instance do we obtain a FPR larger than 1% for DESeq (2% for edgeR) – (see Figures 1 and 3A). It is worth mentioning that the latest updates to DESeq (v1.6.1) and edgeR (v2.4.0) – released after the studies [37] and [26] reduce the number of false positive calls by about 50% (data not shown).

### Effects of replication for detection of DE

To quantify the effects of replication in RNA-Seq DE experiments, we tested *n*-control vs. *n*-treatment biological replicates (2, 3, 4, 6, 8 and 12) while maintaining sequencing depth constant. We find that as *n* increases both algorithms increase the call rate and TPR while the FPR remains unchanged (Table 1).

Our results clearly support the simple message that more biological replicates are not only desirable but needed to improve the quality and reliability of DE detection using RNA-Seq, however, due to the costs associated with RNA-Seq, many experiments are likely to need to use multiplex designs to achieve this level of replication.

This study is concerned with the simulation of overdispersion effects due to biological variability and it is implied that overdispersion due to technical variability is nested within this estimation (see Methods section). It is worth mentioning that, while biological variability is important, the contribution to overdispersion by technical variation is not negligible, and disagreements between estimates of expression can occur at all levels of coverage [41]. Ideally, RNA-Seq experimental design with biological replication should also aim to block sources of technical variation, such as between lane variations, to constrain the dispersion of RNA-Seq experiments.

### Effects of sequencing depth for detection of DE

To quantify the effects of sequencing depth in RNA-Seq DE experiments, we simulated an extensive sequencing depth range (100% to 1%) for every case of *n*-control vs. *n*-treatment biological replicates. As the amount of available sequencing data is decreased, both packages decrease the call rate and TPR while the FPR remains low. TPR decreases very slowly as sequencing depth decreases, suggesting that sequencing depth can be reduced to ∼15*%* without much impact on TPR.

We conclude that DE analysis with RNA-Seq is robust to substantial loss of sequencing data as indicated by a slow decline in TPR as sequencing depth is lost accompanied by no increase in FPR. These findings seem consistent with the results reported by Bashir et al. [42] who observed that lower levels of transcriptome sequencing had sufficient information to estimate the distribution of expression values arising from observed transcripts. Bashir et al. did not directly test power to detect DE, however as testing for DE relies on good concordance with the expected distribution, it follows that DE is reasonably robust to loss of sequencing data.

### Multiplexing experimental designs

To quantify the effects of varying both *n* and sequencing depth, we simulated multiplexing *n*-control vs. *n*-treatment lanes into two sequencing lanes. We observed a steady increase in TPR with the increase in *n*, despite the corresponding decrease in sequencing data per transcript by 1/*n*. Similarly, for both DESeq and edgeR the number of DE calls and the TPR increases with *n*, as we observed previously and is unaffected by the decrease in data. For DESeq, the FPR remains roughly constant and always below 0.1%, while for edgeR, the FPR decreases slowly as *n* increases.

Our simulations strongly support that the benefits of multiplexing *n*-biological replicates into one sequencing lane (two lanes for a *n*-control vs. *n*-treatment DE experiment), far outweigh the decrease of available data per sample by 1/*n*. These multiplexing experimental designs improve TPR and FPR while greatly reducing the cost of the experiment.

While the detection of DE appears robust to available sequence data, there remains the question of how multiplexing affects coverage of the transcriptome and detection of low abundant or rare transcripts. This coverage issue will increasingly be counterbalanced by rapid increases in data generation capacity from a single sequencing experiment. In a detailed study of the Marioni [23] human (liver and kidney) dataset, Banshir et al. [42], reported that over 90% of the total observed transcripts were sampled with 1 million reads. This should be considered in the context of the quickly evolving sequencing technologies like HiSeq 2000 and HiSeq 2500 which can produce up to 300 million reads per sequencing lane. In an evaluation of coverage depth of the chicken transcriptome, Wang et al. [21] find that while 10 million reads allow detection of 80% of the annotated genes, an increase from 10 to 20 million reads does not have a significant effect on transcriptome coverage or reliability of mRNA measurements. That said, current estimates of transcriptome coverage and the impacts of multiplexing strategies analysed in this paper assume unbiased sampling of transcripts. It is highly likely that the power to detect DE varies across transcripts with their sequence content, isoform complexity and abundance. Fang and Cui [20] warn against and discuss several sequencing biases that could create the need for high sequencing coverage to accurately estimate transcript abundance and variation. The authors mention the importance of choosing whether to increase the sequencing depth per sample or to increase the number of biological replicates when planning an experiment. Here, we quantitatively argue that given a fixed budget, the benefits of increasing the number of biological replicates outweigh the corresponding decrease of sequencing depth. This suggestion is backed by the patterns in Figures 3 and 4 in which for a given number of *n*-biological replicates TPR drops very slowly as depth decreases, FPR remains low when sequencing depth is decreased. In the light of new sequencing technologies rapidly increasing the available sequencing depth per lane, the inforrmation provided by biological replicates’ variation is likely to become a priority over sequencing depth.

## Conclusions

Not surprisingly, our results indicate that more biological replicates are needed to improve the quality and reliability of DE detection using RNA-Seq. Importantly however, we also find that DE analysis with RNA-Seq is robust to substantial loss of sequencing data as indicated by a slow decline in TPR accompanied by no increase in FPR. Our simulations strongly support that multiplexing experimental designs improve TPR and FPR while greatly reducing the cost of the experiment, as the benefits of multiplexing n-biological replicates far outweigh the decrease of available data per sample by 1/*n*.

As many available packages for DE analysis are increasingly becoming faster and easier to use, our recommendation for most RNA-Seq DE experiments is to use 2 different packages for DE testing. Additional file 2: Figure S4 illustrates the detection overlap between DESeq and edgeR for two contrasting choices of sequencing depths and n-biological replicates: n = 2 at 100% depth and n = 4 at 25% depth. The combined use of packages based on different distribution statistics or a different set of assumptions could generate useful information about a possible bias susceptibility of a given package particular to the specific dataset of interest.

To our knowledge, this is the most up-to-date comparison of DESeq and edgeR’s performance relative to ability to detect DE in a range of experimental designs. It directly tests the efficiency of modern multiplex experimental design strategies. Our study informs important experimental design decisions now relevant when trying to maximise an RNA-Seq study to reliably detect DE.

## Methods

### Negative binomial model and biological variation simulation

Our synthetic data is based on a negative binomial (NB) model of read counts assumed by [39] and used in edgeR [32], DESeq [25] and NBPSeq [33]. The model is a hierarchical model which takes into account sources of variability in the molar concentration of each transcript isoform in the prepared cDNA library due to *i)* library preparation steps and, in the case of biological replicates, *ii)* biological variation. This variation is compounded by an additional Poisson shot-noise arising from the sequencing step. Assuming the molar concentration in the prepared cDNA library to have a Gamma distribution, one arrives at a NB distribution for the number of counts *K* mapped onto a particular transcript of interest in a given lane of the sequencer:

(1) K∼NB(mean=μ,var=μ(1+ϕμ)).

The mean *μ* is proportional to the concentration of the transcript of interest in the original biological sample, up to a normalisation factor specific to the lane of the sequencer. A suitable model for this normalisation factor is the Robinson-Oshlack TMM factor [32]. The quantity *ϕ *is called the dispersion parameter [39], and is specific to the transcript isoform and the library preparation. A more detailed account of the model is given in the Additional file 3.

### R packages for DE in RNA-Seq

All three packages considered are based on a NB model, and differ principally in the way the dispersion parameter is estimated. Unless otherwise stated, tests of these packages used herein use default settings. Typical coding sequences are given in the Additional file 3.

#### edgeR (version 2.4.0, Bioconductor)

To begin with, edgeR [43] calculates for each transcript a quantile adjusted log conditional likelihood function for the dispersion *ϕ*[39]. Here, “quantile adjusted” refers to an adjustment of the number of counts to adjust for the total number of counts across all transcripts in each biological replicate, and “conditional” means conditioning on the sum of counts for the given transcript across biological replicates. The “common dispersion” estimate defined by edgeR assumes *ϕ*to be a constant over all transcripts in one lane of the sequencer, and is obtained by maximising the log-likelihood summed over transcripts. However, edgeR recommends a “tagwise dispersion” function, which estimates the dispersion on a gene-by-gene basis, and implements an empirical Bayes strategy for squeezing the estimated dispersions towards the common dispersion. Under the default setting, the degree of squeezing is adjusted to suit the number of biological replicates within each condition: more biological replicates will need to borrow less information from the complete set of transcripts and require less squeezing.

#### DESeq (version 1.6.1, Bioconductor)

In previous versions of the package DESeq [25], *ϕ *was assumed to be a function of *μ *determined by nonparametric regression. The recent version used in this paper follows a more versatile procedure. Firstly, for each transcript, an estimate of the dispersion is made, presumably using maximum likelihood. Secondly, the estimated dispersions for all transcripts are fitted to the functional form:

(2) $$\phi =a+\frac{b}{\mu }\;\text{(DESeq parametric fit)},$$

using a gamma-family generalised linear model. The per-transcript estimate is considered to be more appropriate when large numbers of replicates (≥ 4) are present, while the functional form is considered to be more appropriate when small numbers of replicates (≤ 2) are present, in which case information is borrowed from the general trend of all transcripts. Recognising that the dispersion may be underestimated by the functional fit, leading to an overestimate of significance in detecting DE, DESeq by default chooses the maximum of the two methods for each transcript. Also by default, DESeq assumes a model in which the mean *μ* differs between conditions, but the dispersion *ϕ *is common across all conditions.

#### NBPSeq (version 0.1.4, CRAN)

As for edgeR, the package NBPSeq [33] considers per-transcript log likelihood conditioned on the sum of counts across replicates. However, NBPSeq further imposes the following functional relationship between *ϕ* and *μ*:

(3) $$\phi =c\mu^{\alpha -2}\;\text{(NBPSeq model)},$$

that is, a linear relationship between log*ϕ *and log*μ*. The cases *α *= 1 and *α *= 2 (equivalent to common dispersion) of this function are referred to as NB1 and NB2 respectively. The global parameters *α* and *c* are estimated by maximising the log conditional likelihood summed over all replicates and transcripts.

### Construction of the synthetic datasets

Each of our synthetic datasets consists of a ‘control’ dataset of read counts $K_{\text{ij}}^{\text{contr}}$ and a ‘treatment’ dataset of read counts $K_{\text{ij}}^{\text{treat}}$, for *i *= 1,…,*t* transcript isoforms sequenced from *j *= 1,…,*n *biological replicate cDNA libraries.

For each transcript isoform, we begin by providing a pair of NB parameters ${\hat{\mu }}_{i}$ and ${\hat{\phi }}_{i}$. A read count $K_{\text{ij}}^{\text{contr}}$ for each isoform in each biological replicate is then generated by sampling randomly from a NB distribution with these estimated parameters to from the control dataset. To create the treatment dataset, the set of isoforms is first divided into a non-regulated subset, an up-regulated subset and a down-regulated subset. A regulating factor *θ*_*i *_= 1,…,*t*, which is equal to 1 (non-regulated), > 1 (up-regulated) or < 1 (down-regulated) is then chosen from a suitable distribution. A treatment read count $K_{\text{ij}}^{\text{treat}}$ is then generated for each isoform in each biological replicate from a NB distribution with the mean $\theta_{i}{\hat{\mu }}_{i}$ and unchanged dispersion ${\hat{\phi }}_{i}$.

The basis for the parameters ${\hat{\mu }}_{i}$ and ${\hat{\phi }}_{i}$ is a subset of the Pickrell [24] dataset of sequenced cDNA libraries generated form mRNA from 69 lymphoblastoid cell lines derived from Nigerian individuals as part of the International HapMap Project. For each individual, a library prepared for the Illumina GA2 platform was split into two portions, with one portion sequenced at the Argonne sequencing centre and the other at the Yale sequencing centre. For 12 of the individuals a second library was also prepared, split, and sequenced at both centres. Only data from the initial 69 libraries sequenced at Argonne was used for the current study. The raw reads were re-aligned onto the human transcriptome (hg18, USCS) using the KANGA aligner [44]. The total number of reads mapped to annotated genes per lane varied substantially from 2 × 10^6^ to 20 × 10^6^. To provide a uniform set of biological replicates from which to estimate ${\hat{\mu }}_{i}$ and ${\hat{\phi }}_{i}$, a subset of 44 libraries for which the total number of mappings to the transcriptome per lane was in the range 10 × 10^6^ to 16 × 10^6^ was chosen. Finally, any transcript for which the total number of reads was less than 44, i.e. an average of less than one transcript per lane, was culled from the dataset to leave a list of 46,446 transcripts. The resulting subset of the Pickrell dataset is considered to exhibit overdispersion due to both library preparation and biological variation.

Note that for generation of synthetic data it is not necessary to provide an accurate estimate of *μ*_*i*_ and *ϕ*_*i*_ for each isoform in the reduced Pickrell dataset, but simply to provide a plausible distribution of values of these parameters over the transcriptome representing typical isoform abundances and their variation due to technical and/or biological overdispersion. Parameter values ${\hat{\mu }}_{i}$ and ${\hat{\phi }}_{i}$, were obtained from the reduced Pickrell dataset as follows. The total number of counts from each of the 44 lanes was first reduced to that of the lane with the smallest number of counts by sampling from the counts in each lane while keeping track of the transcript to which each count is mapped. This forms a normalised set of counts for the *i*th transcript in the *j*th lane.

For each transcript a maximum likelihood estimate (MLE) ${\hat{\mu }}_{i}$ and ${\hat{\phi }}_{i}$, was then made from the *n *= 44 biological replicates. Details of the construction of this estimate are given in the Additional file 3. For each simulation described herein, a synthetic dataset was constructed consisting of *n* biological replicates of ‘control data’ generated from NB distributions with the estimated ${\hat{\mu }}_{i}$ and ${\hat{\phi }}_{i}$, and a further *n* biological replicates of treatment data generated from NB distributions with means $\theta_{i}{\hat{\mu }}_{i}$ and unchanged dispersion ${\hat{\phi }}_{i}$.

Two sets of simulations were performed: 

1. To test performance under the null hypothesis, the regulating factor was set to *θ*_*i *_= 1 for all transcripts.

2. To test ability to detect DE in the presence of an alternative hypothesis, the regulating factor *θ*_*i *_was set to 1 + *X*_*i *_for a randomly chosen 7.5% of the transcripts (up-regulated), (1 + *X*_*i*_)^−1^ for a further 7.5% (down-regulated) and 1 for the remaining 85% of the transcripts, where the *X*_*i *_are identically and independently distributed exponential random variables with mean 1.

### Calculation of true and false positive rates

#### Under the null hypothesis

All three packages test for DE in single-factor experiments by calculating p-values using the method described in [25]. For each transcript *i*, a probability is calculated for the number of counts in each of two conditions control and treatment, conditional on the sum of the counts in both conditions assuming the NB model described above. The p-value is the sum of the probabilities of all ways of apportioning the sum of counts between the two conditions, which have a lower probability than the observed counts.

To test the performance of each package under the null hypothesis, we simulated sets of *n*-control and *n*-treatment lanes of counts for a range of values of *n*. The FPR, quoted as a percentage, was calculated at a given significance level *α* as:

(4) $$\begin{array}{c} \text{FPR}=\frac{\text{number of transcripts with}\;100\times \text{p-value}<\alpha }{\text{total number of transcripts}}\times 100\mathrm{\%.} \end{array}$$

Ideally, the FPR should match the significance level of *α* if the package is performing correctly.

#### In the presence of an alternative hypothesis

All three packages provide an adjusted p-value, *p*_adj_, to correct for multiple hypothesis testing with the Benjamini-Hochberg procedure using the R function p.adjust(). All calculations herein of true and false positive rates in the presence of an alternative hypothesis use adjusted p-values.

From the 6,966/46,446 (15%) of the transcripts induced with a regulating factor other than 1, we selected the 5,726 (12%) with a regulation factor satisfying either *θ*_*i *_≤ 0.83 or *θ*_*i *_≥ 1.20. We define these as “effectively DE” transcripts. This additional filter on minimal fold-change is designed to quantify the performance of algorithms and experimental designs for detection of DE that might be considered more biologically relevant by researchers. Likewise we define the remaining transcripts, those satisfying 0.83 <* θ*_*i *_< 1.20, as “effectively non-DE”. These definitions were used to estimate the FPR and TPR at significance level *α* via the following formulae:

(5) $$\begin{array}{c} \text{FPR}\;=\;\frac{\text{number of effectively non-DE transcripts with}\;100\times p_{\text{adj}}\;<\alpha }{\text{total number of effectively non-DE transcripts}}\;\;\;\times \;\;\;100\mathrm{\%.} \end{array}$$

(6) $$\begin{array}{c} \text{TPR}=\frac{\text{number of effectively DE transcripts with}\;100\times p_{\text{adj}}\;<\alpha }{\text{total number of effectively DE transcripts}}\times 100\mathrm{\%.} \end{array}$$

Apart from the use of adjusted p-values, the formula for FPR reduces to Eq. 4 if the number of simulated DE transcripts is set to zero, since in this case all transcripts are, by definition, “effectively non-DE”. The quantities 1−FPR and TPR are commonly referred to in the literature as “specificity” and “sensitivity” respectively.

### Simulating variable levels of sequence data and replication

Simulating variations in available sequencing data is a fundamental part of investigating the impacts of multiplex experimental design strategies. Variability in the amount of sequence data amongst samples can occur for reasons such as restrictions on available resources, machine error, or sequencing reads sequestered by pathogen transcriptome fractions present in the sample. To simulate loss of sequencing depth, we randomly sub-sampled without replacement counts from the original table of counts simulated in the presence of an alternative hypothesis for each biological replicate. Sequencing depth was decreased in both control and treatment samples over a range of 100% (a full lane of sequence) to 1% of the original data. After this sub-sampling, the resulting table of counts was analysed in DESeq (edgeR) and the total number of effectively-DE calls, TPR, FPR and fold-changes were recorded for every *n* scenario. We simulated experimental choices of *n*-controls vs. *n*-treatments biological replicates at *n *= 2, 3, 4, 6, 8 and 12.

### Multiplexing experimental designs

Multiplexing various samples into one sequencing lane reduces the monetary cost of RNA-Seq DE analysis, albeit by dividing the available sequencing depth over various samples. Our strategy consisted of simulating multiplexing *n*-control samples vs. *n* treatment samples into two sequencing lanes. This way, the amount of total sequenced data is constrained and each control and treatment sample is expected to be represented at an average depth 1/*n*×100*%*. The absolute value of reads produced in a lane of sequence (i.e. 100% depth) has increased as RNA-Seq technologies evolve, currently this value can be up to 100 million reads. The multiplex experimental setups we tested are: 

• 2 vs. 2 biological replicates at 50% sequencing depth

• 3 vs. 3 biological replicates at 33% sequencing depth

• 4 vs. 4 biological replicates at 25% sequencing depth

• 6 vs. 6 biological replicates at 17% sequencing depth

• 8 vs. 8 biological replicates at 13% sequencing depth

• 12 vs. 12 biological replicates at 8% sequencing depth

• 32 vs. 32 biological replicates at 3% sequencing depth

• 96 vs. 96 biological replicates at 1% sequencing depth

## Endnotes

^a^Our results obtained using edgeR are presented in the Additional file 1: Figure S2.

^b^Additional file 4: Figure S3 shows the minimum fold-change at which a transcript is assigned a *p*_adj_ ≤ 0.01 for every *n* scenario.

^c^The details of our negative binomial model can be found in Additional file 3, including Additional file 5: Figure S1, which shows the maximum likelihood estimates of the model’s mean and dispersion parameters for 46, 446 transcript isoforms.

## Competing interests

The authors declare that they have no competing interests.

## Authors’ contributions

All authors contributed to the manuscript. JAR, SEQ and SJS performed the statistical and bioinformatic analysis. CJB developed the data simulation algorithm. CJB, SRW and JMT conceived and designed the study. All authors read and approved the final manuscript.

## Supplementary Material

Additional file 1 **Figure S2.** FPR and TPR detected by edgeR as a function of sequencing depth and replication. Different symbols represent the number *n* of control vs. treatment samples (*n *= 2, 3, 4, 6, 8, and 12) across sequence depths [100*%*→1*%*]. **A:** TPR *p*_adj_ ≤ 0.01. **B:** FPR *p*_adj_ ≤ 0.01. The solid grey line (“multiplex line”) connecting the TPR values of *n* biological replicates at $\frac{1}{n}\times 100\%$ sequencing depth shows the increase of TPR as more biological replicates *n* are used despite the loss power due to the sequencing depth reduction required by the multiplexing of lanes. This trend remains true even for the *n *= 32 and *n *= 96 cases. Click here for file

Additional file 2 **Figure S4.** Venn-diagram showing the TP and FP calls made by DESeq (left, blue circle) and edgeR (right, red circle) and how they overlap between each other and the total pool of transcripts designated as truly DE (top, green circle). **A:** the Venn-diagram for the case in which the number of biological replicates is *n *= 12 and depth is 100%. This combination of *n* and depth is somewhat unrealistic as the cost of 24 lanes of sequencing would be almost prohibitive; however, it shows a ‘best case scenario’ situation in which 2870 of the total 5689 truly DE transcripts were detected by the union of DESeq and edgeR. Of these 2870 TP detections, most of them (2360) were detected by both algorithms – hence either algorithm would have sufficed. **B:** the Venn-diagram for *n *= 4 and depth is 25%. This more realistic experimental design choice of *n* and depth shows the value of using both algorithms; only 913 out of the total 5697 truly DE transcripts were detected by both algorithms, only two thirds of them (591) were detected by both algorithms. These contrasting scenarios show that the use of both algorithms aids to further constrain the list of viable DE candidates in a fast and cheap manner. Click here for file

Additional file 3 **Negative binomial model**^**c**^**.** Click here for file

Additional file 4 **Figure S3.** Smallest fold-change required for a transcript to be called DE (*p*_adj_ ≤ 0.01) as a function of *n* biological replicates (using DESeq). The more replicates available; the smaller the fold-change required for a transcript to be called DE by DESeq or edgeR. Click here for file

Additional file 5 **Figure S1.** Maximum likelihood estimates of the NB mean ${\hat{\mu }}_{i}$ and dispersion parameter $\hat{\phi }$ for 46,446 transcript isoforms. The green line is a linear regression of $\log_{10}\hat{\phi }$ against $\log_{10}\hat{\mu }$, and corresponds to the NBPSeq model relationship $\hat{\phi }=c{\hat{\mu }}^{\alpha -2}$ with *α *= 1.700, *c *= 0.364. Click here for file
