# Supplementary figures and images for: Efficient experimental design and analysis strategies for the detection of differential expression using RNA-Sequencing
Source: BMC Genomics. 2012 Sep 17;13:484. doi: 10.1186/1471-2164-13-484 (PMC3560154; doi:10.1186/1471-2164-13-484)

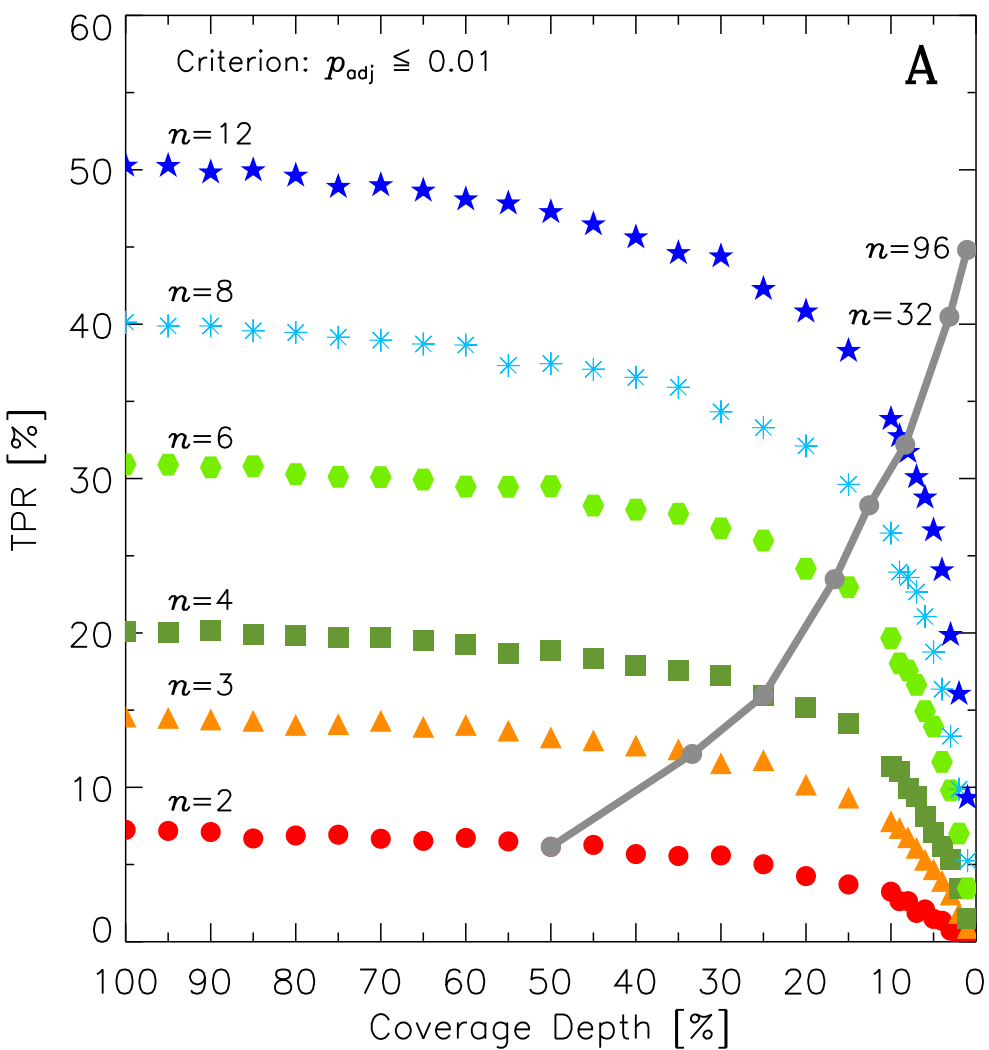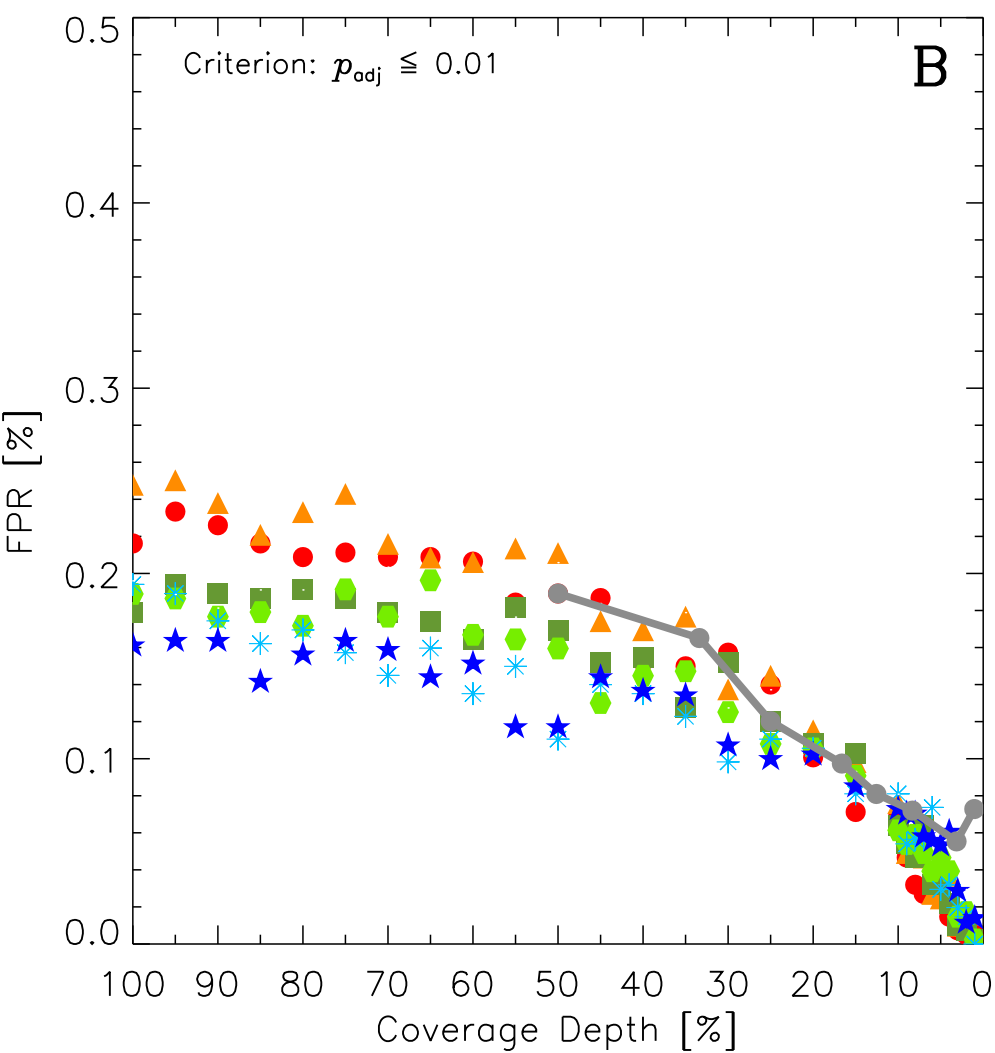

Supplement: Additional file 1 — Figure S2. FPR and TPR detected by edgeR as a function of sequencing depth and replication. Different symbols represent the number n of control vs. treatment samples (n = 2, 3, 4, 6, 8, and 12) across sequence depths [100%→1%]. A: TPR padj ≤ 0.01. B: FPR padj ≤ 0.01. The solid grey line (“multiplex line”) connecting the TPR values of n biological replicates at 1n×100% sequencing depth shows the increase of TPR as more biological replicates n are used despite the loss power due to the sequencing depth reduction required by the multiplexing of lanes. This trend remains true even for the n = 32 and n = 96 cases. [file 1471-2164-13-484-S1.pdf]

n=12, depth=100%

**A**

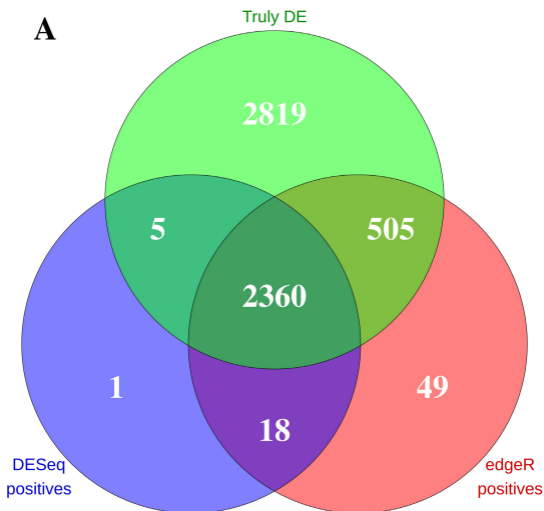

n=4, depth=25%

**B**

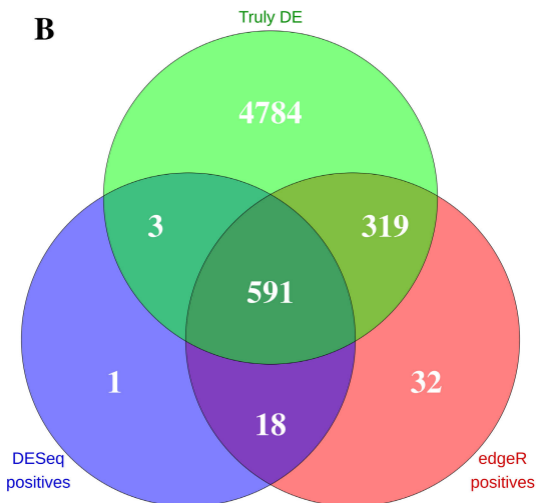

Supplement: Additional file 2 — Figure S4. Venn-diagram showing the TP and FP calls made by DESeq (left, blue circle) and edgeR (right, red circle) and how they overlap between each other and the total pool of transcripts designated as truly DE (top, green circle). A: the Venn-diagram for the case in which the number of biological replicates is n = 12 and depth is 100%. This combination of n and depth is somewhat unrealistic as the cost of 24 lanes of sequencing would be almost prohibitive; however, it shows a ‘best case scenario’ situation in which 2870 of the total 5689 truly DE transcripts were detected by the union of DESeq and edgeR. Of these 2870 TP detections, most of them (2360) were detected by both algorithms – hence either algorithm would have sufficed. B: the Venn-diagram for n = 4 and depth is 25%. This more realistic experimental design choice of n and depth shows the value of using both algorithms; only 913 out of the total 5697 truly DE transcripts were detected by both algorithms, only two thirds of them (591) were detected by both algorithms. These contrasting scenarios show that the use of both algorithms aids to further constrain the list of viable DE candidates in a fast and cheap manner. [file 1471-2164-13-484-S2.pdf]

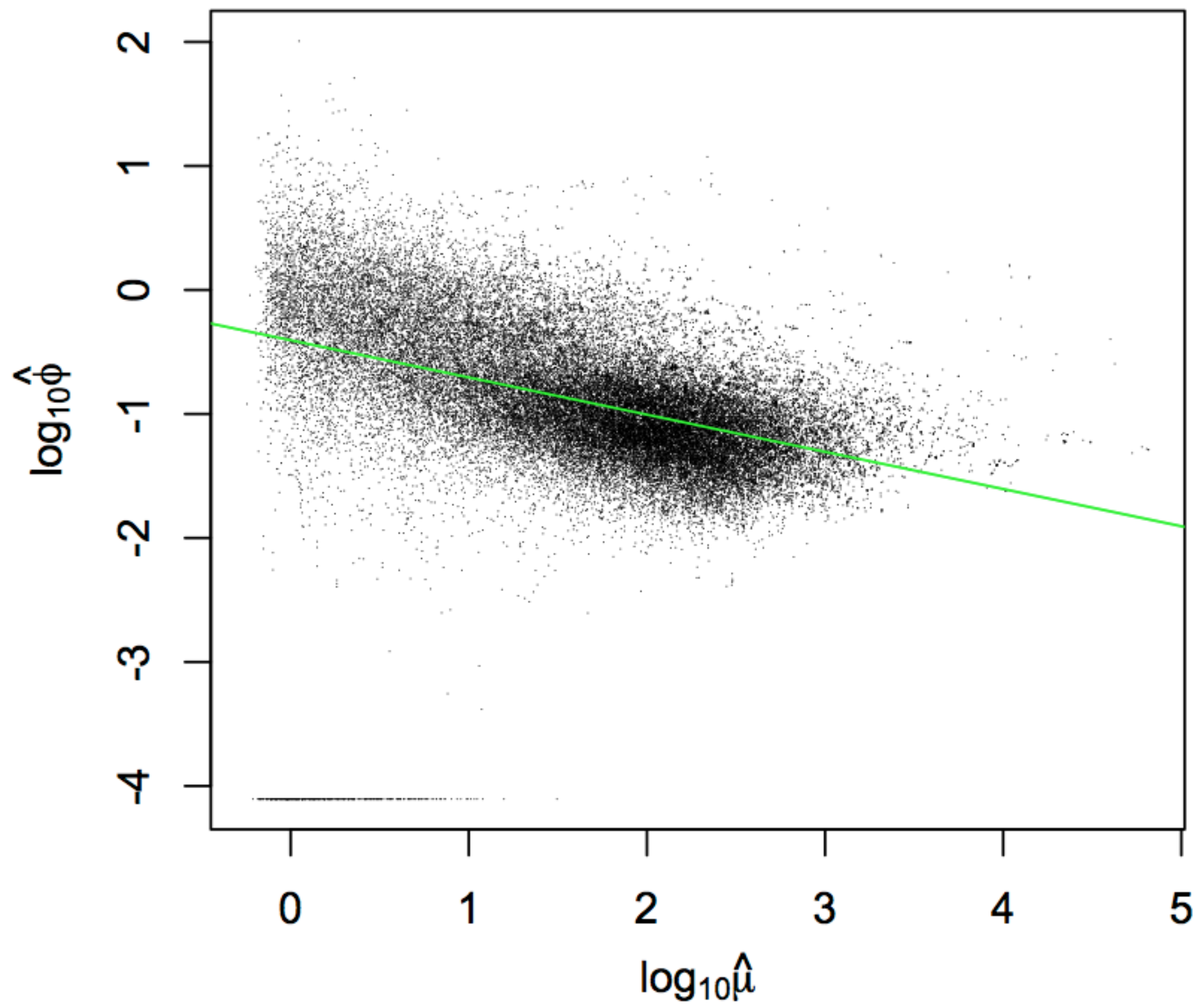

Supplement: Additional file 5 — Figure S1. Maximum likelihood estimates of the NB mean μ^i and dispersion parameter ϕ^ for 46,446 transcript isoforms. The green line is a linear regression of log10ϕ^ against log10μ^, and corresponds to the NBPSeq model relationship ϕ^=cμ^α−2 with α = 1.700, c = 0.364. [file 1471-2164-13-484-S5.pdf]
