# Supplementary material for: Efficient experimental design and analysis strategies for the detection of differential expression using RNA-Sequencing
Source: BMC Genomics. 2012 Sep 17;13:484. doi: 10.1186/1471-2164-13-484 (PMC3560154; doi:10.1186/1471-2164-13-484)
Supplement: Additional file 3 — Negative binomial modelc. [file 1471-2164-13-484-S3.pdf]

Supplementary Material

Negative binomial model

Our synthetic data is based on a negative binomial (NB) model of read counts assumed by Robinson and Smyth [39] and used in edgeR [43], DESeq [25] and NBPSeq [33]. The model is a hierarchical model constructed as follows: Consider one particular lane of the sequencer, and let the abundance of a particular transcript of interest, as measured by its molar concentration in the prepared cDNA library, be the random variable R. Set

$$E(R) = q, \qquad \mathrm{Var}(R) = v. \tag{7}$$

Sources of variability in R include library preparation steps such as fragmentation and size selection of mRNA, reverse transcription to cDNA, and PCR amplification in the case of library-technical replicates, and, in the case of biological replicates, biological variation.

Let the number of reads mapped onto this transcript's gene be the random variable K. Marioni et al. [23] have established experimentally that K conditioned on the cDNA library is accurately modelled by a Poisson distribution. This is to be expected given that the sequencer takes a large sample of reads from an effectively infinite population and a given read will map onto the gene of interest with fixed low probability. Thus we have

$$K|(R = r) \sim \mathrm{Pois}\,(\lambda r), \tag{8}$$

where λ is some suitable normalisation factor for this lane such as the Robinson-Oshlack TMM factor [32]. It follows that

$$E(K|R = r) = \lambda r, \qquad \mathrm{Var}(K|R = r) = \lambda r. \tag{9}$$

As noted by Anders and Huber [25], it follows from Eqs. (7) and (9) using the law of total expectation and the law of total variance that the marginal mean and variance of K are

$$E(K) = \mu, \qquad \mathrm{Var}(K) = \mu(1 + \phi\mu), \tag{10}$$

where

$$\mu = \lambda q, \qquad \phi = \frac{v}{q^2}. \tag{11}$$

More specifically, if we further assume R to be a Gamma random variable, then it is straightforward to show that K is a Negative Binomial random variable. In particular, if

$$R \sim \mathrm{Gamma}(\mathrm{mean} = q, \mathrm{var} = v), \tag{12}$$

so that

$$\lambda R \sim \text{Gamma}(\text{mean} = \mu, \text{var} = \phi\mu^2), \tag{13}$$

then

$$K \sim \text{NB}(\text{mean} = \mu, \text{var} = \mu(1 + \phi\mu)). \tag{14}$$

The NB distribution probability mass function is given by

$$\Pr(K = k|\mu, \phi) = \frac{\Gamma(k + \phi^{-1})}{\Gamma(k+1)\Gamma(\phi^{-1})} \frac{(\mu\phi)^k}{(1 + \mu\phi)^{k+\phi^{-1}}}. \tag{15}$$

The quantity ϕ is called the dispersion parameter [39]. In the limit $\phi \to 0$ the NB distribution reduces to a Poisson distribution with mean μ.

Estimate of the parameters $\hat{\mu}_i$ and $\hat{\phi}_i$

For each transcript isoform in our synthetic dataset, we begin by providing a pair of NB parameters $\hat{\mu}_i$ and $\hat{\phi}_i$. The basis for these parameters is a subset of the Pickrell [24] dataset of sequenced cDNA libraries generated from mRNA from 69 lymphoblastoid cell lines derived from Nigerian individuals as part of the International HapMap Project. Of these a subset of 44 libraries for which the total number of mappings to the transcriptome per lane was in the range 10.0 to 16.0×10^6 was chosen. Finally, any transcript for which the total number of reads was less than 44, i.e. an average of less than one transcript per lane, was culled from the dataset to leave a list of 46,446 transcripts. The resulting subset of the Pickrell dataset is considered to exhibit overdispersion due to both library preparation and biological variation.

Note that for the purpose of the current exercise it is not necessary to provide an accurate estimate of μ_i and ϕ_i for each isoform in the reduced Pickrell dataset, but simply to provide a plausible distribution of values of these parameters over the transcriptome representing typical isoform abundances and their variation due to technical and/or biological overdispersion. Parameter values $\hat{\mu}_i$ and $\hat{\phi}$ were obtained from the reduced Pickrell dataset as follows. The total number of counts from each of the 44 lanes was first reduced to that of the lane with the smallest number of counts by sampling from the counts in each lane while keeping track of the transcript to which each count is mapped. This forms a normalised set of counts K_{ij}^{Pickrell} for the ith transcript in the jth lane.

For each transcript a maximum likelihood estimate (MLE) of $\hat{\mu}_i$ and $\hat{\phi}_i$ was then made from the $n_{\text{rep}} = 44$ biological replicates. The log-likelihood function for a set of identically and distributed NB observations

$y_1, \ldots, y_{n_{\text{rep}}}$is, from Eq. (15),

$$\begin{aligned}\ell(\mu, \phi | y_1, \ldots, y_{n_{\text{rep}}}) &= \sum_{j=1}^{n} \log \Pr(Y_j = y_j | \mu, \phi) \\ &= \sum_{j=1}^{n} \log \Gamma(y_j + 1/\phi) - n \log \Gamma(1/\phi) - \sum_{j=1}^{n} \log \Gamma(y_j + 1) \\ &\quad + \sum_{j=1}^{n} y_j \log \left(\frac{\mu\phi}{1 + \mu\phi} \right) - \frac{n}{\phi} \log (1 + \mu\phi) . \end{aligned} \tag{16}$$

This log-likelihood function differs from that used by edgeR and NBPSeq in that it is not conditioned on the sum of counts across replicates. From Eq. (16) one easily confirms that the MLE of μ_i is given by the usual formula for an unbiased estimates of a mean,

$$\hat{\mu}_i = \frac{1}{n_{\text{rep}}} \sum_{j=1}^{n_{\text{rep}}} K_{ij}^{\text{Pickrell}}. \tag{17}$$

The MLE of ϕ_i for each transcript is then found by numerically maximising the log-likelihood function, with y_j set equal to the observed counts K_{ij}^{Pickrell}. The set of parameters for the 46,446 transcript isoforms are plotted in Additiona file 5: Figure S1.

Software versions and coding sequences

Below we list the software versions used throughout our analyses:

- R 2.14.0

  (`www.r-project.org`)

- Biobase Bioconductor 2.14.0

  (`www.bioconductor.org`)

- edgeR 2.4.0

  (`bioconductor.org/packages/release/bioc/html/edgeR.html`)

- DESeq 1.6.1

  (`bioconductor.org/packages/release/bioc/html/DESeq.html`)

- NBPSeq 0.1.4

  (`http://cran.r-project.org/web/packages/NBPSeq/index.html`)

- Python 2.7.2+

  (`www.python.org`)

When testing for DE using edgeR, DESeq and NBPSeq we used the packages default commands Typical coding sequence used in our simulations for detecting differential expression between two conditions, A and B, with n_{rep} biological replicates in each condition, are as follows:

edgeR (version 2.4.0)

```
> conds <- c(rep("A",nrep), rep("B",nrep))
> dgl <- DGEList(counts=Data, group=conds,
                 lib.size=colSums(Data))
> dgl <- calcNormFactors(dgl, refColumn=1)
> dgl <- estimateCommonDisp(dgl)
> dgl <- estimateTagwiseDisp(dgl)
> result <- exactTest(dgl, dispersion="tagwise")
```

If Benjamini-Hochberg(BH)-adjusted p-values are required, the final line of code is replaced by

```
> res <- exactTest(dgl, dispersion="tagwise")
> result <- topTags(res, n=nrow(res),
             adjust.method="BH", sort.by="p.value")
```

DESeq (version 1.6.1)

```
> conds <- factor (c(rep("A",nrep), rep("B",nrep)))
> cds <- newCountDataSet(Data, conds)
> cds <- estimateSizeFactors(cds)
> cds <- estimateDispersions(cds)
> result <- nbinomTest(cds,"A", "B")
```

The final line of code returns both exact p-values and BH-adjusted p-values.

NBPSeq (version 0.1.4)

```
> grp.ids <- c(rep(1,nrep), rep(2, nrep))
> grp1 <- 1
> grp2 <- 2
```

```
> set.seed(999) # to make results reproducible
> NFactor <- dgl$samples$norm.factors # TMM factors
> result <- nbp.test(Data, grp.ids, grp1, grp2,
                     norm.factors=NFactor,
                     method.disp = "NBP")
```

This code uses TMM normalisation factors [32] from the `DGEList` object evaluated in edgeR.
